# Cerebrospinal fluid from patients with amyotrophic lateral sclerosis inhibits sonic hedgehog function

**DOI:** 10.1371/journal.pone.0171668

**Published:** 2017-02-07

**Authors:** Anna Drannik, Joan Martin, Randy Peterson, Xiaoxing Ma, Fan Jiang, John Turnbull

**Affiliations:** 1 Department of Medicine, Division of Neurology, McMaster University, Hamilton, Ontario, Canada; 2 Life Sciences Services, SGS Life Sciences Services Canada Mississauga, Ontario, Canada; Children's Hospital of Pittsburgh, University of Pittsburgh Medical Center, UNITED STATES

## Abstract

Sonic hedgehog (Shh) is a morphogen essential to the developing nervous system that continues to play an important role in adult life by contributing to cell proliferation and differentiation, maintaining blood-brain barrier integrity, and being cytoprotective against oxidative and excitotoxic stress, all features of importance in amyotrophic lateral sclerosis (ALS). ALS is a fatal disease characterized by selective loss of motor neurons due to poorly understood mechanisms. Evidence indicates that Shh might play an important role in ALS, and that Shh signaling might be also adversely affected in ALS. Since little is known about the functional status of Shh pathway in patients with ALS, we therefore sought to determine whether Shh protein levels or biological activity in cerebrospinal fluid (CSF) was less in ALS patients than controls, and whether these measures could be correlated with ALS disease severity and disease progression, and with other CSF analytes of biological interest in ALS. Comparing Shh levels in the CSF of normal controls (n = 13), neurological controls (n = 12), and ALS patients (n = 9) measured by ELISA, we found that CSF Shh levels were not different between controls and ALS patients. However, when assessing Shh biological activity in CSF using *in vitro* cell-based assays, which measure Shh activity as inducible Gli-driven luminescence, we found that in the presence of exogenous recombinant Shh or the Shh agonist, purmorphamine, the inducible activity of CSF was significantly augmented in the control groups as expected, but not in the ALS group, suggesting the presence of an inhibitor of Shh signaling in ALS CSF samples. Since purmorphamine acts on Smoothened, downstream of Shh and its receptor Patched, the inhibitory action is downstream of Smoothened. Our results also demonstrated that while the inhibitory effect of ALS CSF on Shh signaling did not correlate significantly with ALS disease characteristics, the levels of IL-1β and TNF-α did. In addition to being significantly elevated in ALS CSF, these cytokines negatively correlated with the disease duration, whereas GDF11 was a favorable predictor of ALS clinical score. We also found that TNF-α significantly inhibited Shh biological activity *in vitro*, potentially suggesting a novel role of TNF-α in ALS pathogenesis. Collectively, this is the first report demonstrating that Shh signaling in CSF of ALS patients is compromised.

## Introduction

ALS is a progressive and ultimately fatal neurodegenerative disease with obscure pathogenesis and no cure. ALS is characterized by selective loss of motor neurons in the spinal cord and motor cortex, leading to progressive weakness of voluntary muscle and death within 2–5 years due to respiratory compromise as reviewed in reference [1].

Several pathological processes have been implicated in the dysfunction and death of motor neurons in ALS, including impaired axonal transport [2], distal axonopathy [3, 4], glutamate excitotoxicity [5, 6], altered trophic support [2], apoptosis [2], mitochondrial dysfunction [7], oxidative stress [8], proteinopathy with accumulation of misfolded proteins [9–12], altered RNA processing [13, 14], loss of blood-brain barrier integrity [15, 16], loss of nuclear import function [17–19], and neuro-inflammation [3, 20, 21]. Although there may be a final common pathway to cell death in ALS, which remains to be discovered, all of the above processes may contribute to the pathogenesis.

Sonic hedgehog (Shh) is a highly conserved morphogen and a pleiotropic molecule of the hedgehog family, which modulates several of the above pathways and hence is of interest in ALS. Shh regulates neurogenesis during embryonic development [22–24] and in adulthood [25]. Shh is required in vivo for the generation of functional motor neurons from neural stem cells, and intrathecal transplantation of these cells was shown to delay the onset of clinical signs and prolong survival in SOD1G93A mice [26]. Shh promotes the integrity and immune quiescence of blood-brain barrier (BBB) by decreasing the expression of inflammatory mediators as well as the adhesion and migration of leucocytes [27], features which may be disrupted in ALS [16, 28, 29]. Shh exerts a cytoprotective effect against oxidative stress in *in vitro* models of ALS [30]. Hence, due to its regenerative, anti-inflammatory, and cytoprotective properties, the Shh pathway may be important in ALS.

In health, Shh signaling begins with the autoproteolytic cleavage and lipid modification of Shh precursor. In the canonical Shh pathway, Shh binds the 12-transmembrane transporter-like receptor Patched (Ptch), which normally inhibits the activity of the 7-transmembrane protein Smoothened (Smo). Binding of Shh to Ptch de-represses Smo [31, 32], allowing Shh signaling cascade to be initiated through a series of phosphorylation events as reviewed by Chen and Jiang [33], leading to the activation of transcription factors of Gli (Glioma-associated oncogene homolog) family: Gli1, Gli2, and Gli3. The activated Gli proteins translocate to the nucleus and regulate translation of Shh target genes. The role of each of Gli transcription factors is not fully understood yet; but there is evidence that Gli factors act as bifunctional transcription effectors through proteolytic cleavage and conversion of a full-length activator form into a truncated repressor form as reviewed in reference [34]. Gli1 acts mostly as an activator, whereas Gli2 and Gli3 may act as both repressor and activator under certain conditions [33, 35, 36]. The ratio of activator and repressor forms of Gli proteins shapes the Shh signaling cascade and cellular responses.

The canonical Shh pathway is regulated by a number of mechanisms and at multiple levels [37, 38]. One of the key regulators is the Suppressor of Fused (Sufu) [36, 39]. In the absence of Shh, Sufu holds Gli factors bound in a cytoplasmic complex preventing them from nuclear translocation and translation of target genes [36, 37, 39]. Sufu has also been shown to physically interact with Gli-1 on DNA and form a repressor complex, as well as to impair the Gli-1 nuclear-cytoplasmic shuttling [39]. Cytoplasmic anchoring of Gli factors by Sufu promotes processing, degradation, and turnover of Gli effectors leading to the inhibition of Shh pathway signaling. Targeted disruption of murine Sufu results in a phenotype associated with neural tube defects, continuous activation of Shh pathway and early embryonic death [40]. Furthermore, targeted proteolysis of Sufu in the nucleus, mediated by Fbxl17 (F-box and leucine-rich repeat protein 17) through ubiquitylation, liberates Gli-1 from Sufu and restores proper Shh signal transduction, whereas depletion of Fbxl17 results in impaired Shh signaling and compromised cancer cell proliferation and medulloblastoma tumor growth [41]. In addition to canonical Shh signaling, a non-canonical pathway has been described not involving Gli-mediated transcription, as reviewed elsewhere [42].

Shh signaling may be impaired in ALS. Shh signaling proceeds through the primary cilium, and in cell culture and in transgenic mouse models of ALS, the primary cilium is largely absent from spinal motor neurons [25, 43], the chief cell type affected in the disease, yet is unaffected in other spinal neurons or in motor neurons of WT mice. Overexpression of mutated SOD1, but not wild-type SOD1, reduced signaling in the Shh pathway [30]. Recently, Aronica and colleagues demonstrated that expression of Shh-associated genes was downregulated in motor cortex samples in one of two groups of patients with sporadic ALS compared to controls [44].

Thus, Shh positively modulates several pathways implicated in the pathogenesis of ALS, and Shh signaling may be defective in ALS. For these reasons, we sought evidence that Shh signaling is defective in ALS patients. We determined whether Shh protein levels or biological activity in cerebrospinal fluid (CSF) was less in ALS patients than controls, and whether these measures could be correlated with ALS disease severity and disease progression, and to other CSF analytes of biological interest in ALS. We found that while CSF Shh levels were not different between controls and ALS patients, the biological activity of Shh in ALS CSF samples was significantly inhibited when measured in an *in vitro* assay. This inhibitory activity of ALS CSF, however, did not correlate with ALS functional score and severity, unlike several other analytes detected in the CSF.

## Patients and methods

### Patients

All patients were enrolled on a protocol approved by the Hamilton Integrated Research Ethics Board and gave written consent. All investigations have been conducted according to the principles expressed in the Declaration of Helsinki. Nine ALS patients were recruited from McMaster University Medical Centre ALS Clinic (7 males and 2 females). Twelve patients, 4 males and 8 females, with a variety of non-ALS neurological conditions (‘neurological controls’) were recruited from the Neurology ward at the Hamilton General Hospital, where they were undergoing lumbar puncture as part of planned investigations. Conditions included one each of: Devic’s disease, metastatic tumor, encephalitis, possible meningitis, vascular dementia, hydrocephalus of unknown cause, idiopathic intracranial hypertension, multifocal motor neuropathy, clinically isolated syndrome (possible MS), trigeminal sensory neuropathy, a confusional state, and one without a firm neurological diagnosis. Thirteen ‘normal controls’, 6 males and 7 females, were recruited from St. Joseph’s Hospital, Hamilton, where they were undergoing spinal anesthesia for hip or knee replacement. Since we wanted to correlate Shh levels and activity with CSF cytokine levels, patients were ineligible if they were on corticosteroids at the time of spinal anesthesia. ALS functional status at the time of lumbar puncture was determined using the revised ALS functional rating scale (ALSFRS-R) [45]. The ALSFRS-R assesses function in a number of domains important to ALS function (breathing, swallowing, speech, mobility, etc.) and has a maximum score of 48 (no impairment). The disease duration was estimated as the time in months from symptom onset until the time of lumbar puncture, and the progression rate was estimated as (48 minus the ALSFRS-R score)/disease duration, as described elsewhere [46].

### Specimen collection and preparation

CSF samples were obtained by lumbar puncture following standard procedures and without any complications. By nature of the sample collection protocol, a variable but usually small amount (0.5–1 ml) of CSF was obtained from the patients undergoing spinal anesthesia, and a somewhat larger amount, usually 1–2 ml, was obtained from the other patients. Samples were promptly centrifuged at 1500 rpm (400 g) for 10 min, and the liquid phase was aliquoted and frozen at −80°C until utilization.

### Protein assay

Protein amount was quantified according to the standard protocol using Bradford assay with bovine serum albumin (BSA) (Sigma-Aldrich, Oakville, Ontario) as a standard and Bio-Rad Dye Reagent Concentrate as a protein stain (Bio-Rad Laboratories, Mississauga, Ontario).

### Luminex

Aliquots of CSF liquid phase samples were simultaneously analyzed by multiplexed fluorescent bead-based MILLIPLEX ^®^ MAP immunoassay based on the Luminex ^®^ platform (Millipore, Etobiko, ON) (Cat #MPXHCYTO-60K). Measurements of ten cytokines and chemokines, namely GM-CSF, IFN-γ, IL-6, IL-8, IL-10, IL-17A, IP-10, MCP-1, MIP-1β, and VEGF were determined in two- and fifty-fold diluted CSF samples according to the manufacturers’ instruction. Briefly, 25 μL of assay buffer, 25 μL of sample or standard, and 25 μL of matrix solution were combined with 25 μL of mixed beads added to each well of a 96 well plate and incubated overnight on an orbital shaker at 4°C. Following washing, 25 μL of detection antibody was added to each well and incubated for one hour at room temperature. After incubation, 25 μL of streptavidin-phycoerythrin was added to each well and incubated on an orbital shaker for 30 minutes at room temperature. The plate was washed and 150 μL of sheath fluid was added to each well, and beads were re-suspended on a plate shaker for 5 minutes. The levels of fluorescence in standards, quality controls, and samples were detected with the Perkin-Elmer Luminex/Bio-plex machine (Perkin-Elmer, Woodbridge, ON). Data were subsequently analyzed using the Bio-plex manager 6.0 software (Bio-Rad Laboratories, Inc., Mississauga, ON). Cytokine concentrations were determined by reference to a standard curve generated for each analyte with the Bio-plex manager software using stepwise five-fold dilution of protein standards (3.2, 16, 80, 400 and 2000 pg/ml). The sensitivity of the Milliplex assay ranged from 0.1–9.5 pg/ml for the analytes studied. The intra-assay precision measured as the coefficient of variation for the studied cytokines ranged from 4.5 to 10.4%. The inter-assay precision measured as the coefficient of variation for the studied analytes ranged from 5.8 to 12.6%.

### ELISA assays

In addition to multiplexed bead-based assay, commercial ELISA kits were used to determine concentrations of human Shh (Abcam, ab100639), GDF11 (Elabscience, E-EL-H1908), sTLR2 (R&D Systems, DY2616), TNF-α (R&D Systems) and IL-1β (R&D Systems, DY201-05) according to the manufacturer’s protocol. Standards for each ELISA were re-suspended as per manufacturer’s protocol. Analytes were quantified based on standard curves obtained using a Tecan Safire ELISA reader (MTX Labs Systems Inc.). Cut off limit for IL-1β was 3.91pg/ml; for Shh it was 7.8 pg/ml; and for GDF11, TNF-α and sTLR2 it was 15.6 pg/ml; levels detected below these limits were considered as undetectable.

### Luminescence assays

We used mainly Shh-Light II (Shh-LTII) cells (ATCC CRL-2795/JHU-68 embryo fibroblast cells) [47] (obtained from Cameron Harlow, Johns Hopkins University GRCF BioRepository and Cell Center, June 2011) to measure Shh functional activity. These cells have a stably incorporated Gli-luc reporter with expression of a light-generating firefly luciferase induced in the presence of Shh. They also have a stably incorporated TK-based Renilla luciferase that is constitutively expressed. Shh-LTII cells were propagated in Dulbecco’s modified Eagle’s medium (DMEM) supplemented with 10% fetal bovine serum (FBS), 1% HEPES, 1% l-glutamine (Invitrogen Life Technologies), 1% penicillin-streptomycin (P/S) (Sigma-Aldrich, Oakville, Ontario), Geneticin (0.4 mg/ml G418, Gibco, Life technologies, Burlington, Ontario) and 0.15 mg/ml of Zeocin, Invitrogen, Burlington, ON) at 37°C in 5% CO_2._ For the assay, Shh-LTII cells were seeded 40,000 cells per well and cultured over 24 h or until 95–100% confluence in a 96-well clear bottom white chimney plates (Garnier Bio-One, Mississauga, ON) in a complete culture media DMEM with 10% FBS, 1% HEPES, 1%P/S. Frozen aliquots of debris-free liquid phase of the CSF were thawed and 25 μl of samples were combined with 25 μl of reduced-serum (0.5% calf serum) DMEM with or without the addition of either 0.3 μg/ml of human recombinant Shh (rShh) (Leinco, St. Louis, MO, USA), or the Shh agonist purmorphamine (Purm) (Stemgent, Lexington, MA, USA) (0.3 μM in pure dimethyl sulfoxide (DMSO), final concentration in well), or Shh antagonist, cyclopamine (Sigma-Aldrich, Oakville, ON) (5 μM in DMSO, final concentration in well), and left on Shh-LTII cells for additional 30–48 h. Following incubation, media was removed from the Shh-LTII cells and cell lysates were assayed for firefly and Renilla luciferase activities using a microplate reader (Tecan, Infinite M1000) with Dual-Glo® Luciferase Assay System (Promega, Madison, WI) according to the manufacturer’s protocol. Firefly luminescence (FFL) in reporter assays was normalized using the Renilla luciferase (RL) internal control, and presented in arbitrary units (AU) as luminescence light units (LLU) of FFL divided by LLU of RL. In some graphs AU ratio was computed as fold increase over baseline as AU stimulated/AU unstimulated. A standard curve was generated using recombinant human Shh to show a dose- and Shh-dependent increase in luminescence in Shh-LTII cells as a positive control for the assay for testing of the biological activity of the CSF-present endogenous Shh.

To corroborate findings from Shh-LTII cells, we used NSC-34 cells, a hybrid of mouse neuroblastoma and embryonic spinal cord cells enriched for motor neurons [48]. NSC-34 cells were kindly provided by Dr. Neil Cashman, University of British Columbia (obtained in November of 2007). Cells were cultured in DMEM supplemented with 10% fetal bovine serum (FBS) (Gibco, Cat # 12484–028), 1% HEPES, 1% l-glutamine (Invitrogen Life Technologies), 1% penicillin-streptomycin (P/S) (Sigma-Aldrich, Oakville, ON) at 37°C in 5% CO_2_ and propagated using 0.25% trypsin-EDTA (Gibco, Cat # 25200–056) as described before [48, 49]. For the luminescence assay, NSC-34 cells were transfected (NSC-34-Gli) in 96-well clear bottom white chimney plates (Garnier Bio-One, Mississauga, ON) with Cignal GLI Reporter construct (SABiosciences, Frederick, MD, USA, CCS-6030L) using Opti-MEM Reduced Serum Medium (Invitrogen, Cat # 31985–062) and Attractene Transfection Reagent (Qiagen, Cat # 301005), as per manufacturer’s protocol. Cignal GLI Reporter is a mixture of GLI-responsive firefly luciferase construct and constitutively expressing Renilla luciferase construct. Twenty four hours after the transfection, the medium was replaced by a serum-reduced medium containing 1:1 DMEM/F12 (Ham), 1% FBS, 1% modified Eagle’s medium nonessential amino acids (NEAA), 1% l-glutamine (Invitrogen Life Technologies), and 1% penicillin-streptomycin (P/S) (Sigma-Aldrich, Oakville, ON). Cells were cultured either in medium alone or in a half-diluted pooled (due to limited volumes in each sample, 3–4 individual CSF samples were pooled into one sample, resulting in n = 3 pooled samples per group, with comparable total protein levels across groups) CSF samples from ALS and control groups. Dual-Glo® Luciferase Assay (Promega, Madison, WI) was performed 48–72 hours after culturing cells to measure GLI-reporter activity. Firefly luminescence was normalized using the Renilla luciferase internal control to present data as arbitrary units (AU) as was done for Shh-LTII cells.

For TNF-α experiments, Shh-LTII and NSC-34-Gli cells were seeded and cultured as for the luminescence assay and subsequently treated in the reduced-serum assay medium with human recombinant TNF- α (RnD, Cat # 210-TA-005) alone (0–125 ng/ml) or in the presence of Purm (0.8 μM). Luminescence output was measured 48–72 hours later with Promega.

### Immunofluorescence staining

Transfected NSC-34-Gli cells were cultured on a poly-D-lysine-coated 8-well Nunc Lab-Tek II chamber slide (Sigma-Aldrich, Cat # C7057-1PAK) and treated with serum-reduced medium alone or a half-diluted pooled CSF from Neuro and ALS groups for 72 hours. Following the treatment, cells were fixed, blocked, and stained as described elsewhere [50] with minor modifications. Cells were treated with primary antibodies overnight at 4°C. Primary antibodies include rabbit polyclonal anti-Gli1 (1:100, Abcam, Cat # ab92611), mouse monoclonal anti-Gli2 (1:100, Santa Cruz Biotechnology, Cat # sc-271786), and mouse monoclonal anti-Sufu (1:100, Santa Cruz Biotechnology, Cat #sc-137014). The next day, cells were washed and incubated with secondary antibodies in a blocking solution for 1 hour at room temperature. Secondary antibody alone served as a negative control. Secondary antibodies include Alexa Fluor 568 goat anti-mouse (1:1000, A11031) and Alexa Fluor 488 goat anti-rabbit (1:1000, A11008) (all form Molecular Probes, Carlsbad, USA). Cells were washed and coverslipped with SlowFade Gold anti-fade reagent with DAPI (Molecular Probes, S36939) and examined. Images were acquired using a widefield, inverted microscope EVOS FL Auto (Thermo Fisher Scientific). Nuclear translocation/expression of Gli1, Sufu, and Gli2 proteins was quantified as a relative nuclear intensity analyzed per cell (ImageJ) based on manually delineated nuclear area in a DAPI channel using ROM function in ImageJ.

### Statistical analysis

Data were expressed and presented as mean ± standard deviation (SD). Statistical analysis was performed with either a non-paired Student’s *t* test or a one-way analysis of variance (ANOVA) with Tukey post-*hoc* test, unless stated otherwise. For TNF-α assay, results were analyzed using linear regression with log10-transformed TNF-α concentration to better demonstrate a linear relationship between dependent and independent variables. Spearman’s rank correlation analysis was also performed to correlate various clinical parameters with levels of biological analytes as well as Shh-LTII assay data, where Spearman correlation coefficient *r* and *p* values were generated for each pair. Statistical significance was set at * *p* ≤ 0.05. All statistical analyses were done using Graph Pad Prism 5.

## Results

### Patients

The demographic data for the patients are depicted in Table 1. We were restricted in the choice of normal controls to patients undergoing spinal anesthesia for orthopedic surgery, who were older on average (67.5 ± 8.6 years) than either neurological controls (42.9 ± 17.3 years) or ALS patients (52.3 ± 10.4 years) (*p* = 0.0013, normal controls versus ALS patients). The disease duration in the ALS patients was 12.2 ± 6.6 (SD) months, and as such, patients were early in the disease course. The mean ALSFRS-R score was 44.9 ± 1.8 (out of 48), again reflecting early disease presentation. The CSF total protein levels were not significantly different among the groups, and the cell counts in all samples fell within normal limits.

**Table 1:** Clinical characteristics of study participants.

	Normal controls	Neurological controls	ALS
**Number of subjects**	13	12	9
**Sex, M/F**	6/7	4/8	7/2
**Age at examination (mean ± SD, y)**	67.5 ± 8.6 *	42.9 ± 17.3	52.3 ± 10.4
**Disease duration (mean ± SD, mo)**	NA	NA	12.2 ± 6.6
**ALSFRS-R score (mean ± SD)**	NA	NA	44.9 ± 1.8
**Total protein in CSF (mean ± SD, g/L)**	0.47 ± 0.19	0.56 ± 0.35	0.45 ± 0.16

Patients with amyotrophic lateral sclerosis (ALS); revised amyotrophic lateral sclerosis functional rating scale (ALSFRS-R); cerebrospinal fluid (CSF); not applicable (NA); standard deviation (SD). Statistical analysis was performed using unpaired t-test with * *p* < 0.05 considered significant. Asterisk indicates a statistically significant difference between the normal controls and the ALS group.

### Direct measurement of Shh in CSF reveals comparable protein levels in ALS patients and controls

**Fig 1:**
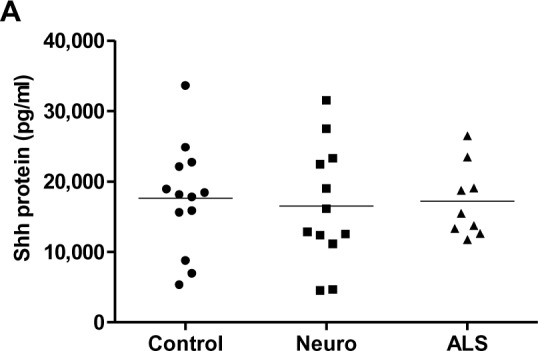
Direct measurement of Shh in CSF reveals comparable protein levels in ALS patients and controls. (A) Shh protein concentrations (pg/ml) in CSF samples are shown in normal controls (Control), neurological controls (Neuro), and patients with ALS (ALS). Protein concentrations were measured by commercial ELISA kit as described in Materials and Methods. Standard was prepared as per manufacturer’s protocol, and Shh protein levels were quantified based on standard curves obtained using ELISA reader. Detected Shh concentrations were17,195 ± 5,155 pg/ml in the ALS group, 17,624 ± 7,676 pg/ml in normal controls, and 16,480 ± 8,495 pg/ml in neurological controls that were not significantly different (*p* = 0.89, normal controls versus ALS).

We measured Shh levels in the CSF by ELISA. Shh protein levels were as follows: 17,195 ± 5,155 pg/ml in the ALS group, 17,624 ± 7,676 pg/ml in normal controls, and 16,480 ± 8,495 pg/ml in neurological controls (Fig 1A). Thus, there were no significant differences in the CSF Shh protein levels (*p* = 0.89, normal controls versus ALS).

### ALS CSF inhibits Shh biological activity *in vitro*

**Fig 2:**
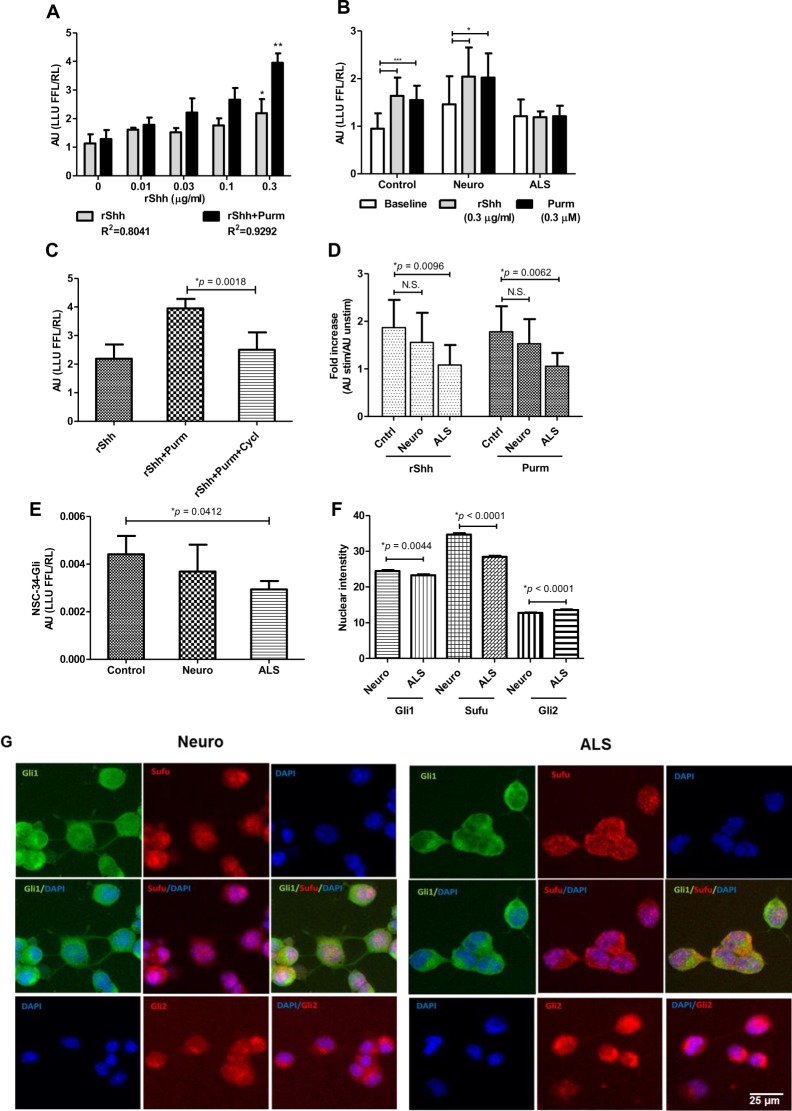
ALS CSF inhibits Shh biological activity *in vitro*. (A) Standard curves with R^2^ demonstrate a dose-dependent response of Shh-LTII cells to human recombinant Shh (rShh) and purmorphamine (Purm) (0.3 μM). Shh-LTII cells were cultured with rShh and Purm and developed with Promega Dual-Glo Luciferase Assay System, assaying for firefly (FFL) and Renilla (RL) luciferase-driven luminescence measured as luminescence light unit (LLU). Data are expressed as mean ± SD and shown as arbitrary units (AU) where FFL was normalized to RL (LLU FFL/RL). * and ** indicate significant differences from media alone for rShh and Purm, correspondingly, based on a one way ANOVA analysis with Tukey post-*hoc* test (*p* < 0.05). (B) The inhibitory effect of ALS CSF on Shh biological activity measured in Shh-LTII cells is shown. Fully confluent Shh-LTII cells were incubated either with CSF alone, half-diluted with reduced-serum medium as a baseline (white bar), or in the presence of rShh (0.3 μg/ml) (grey bar) or Purm (0.3 μM) (black bar) and assayed with Promega. Data are expressed as mean ± SD and shown as AU. * and *** indicate significant differences between a baseline, rShh, and Purm in neurological controls (Neuro) or normal controls (Controls), respectively, using one way ANOVA with Tukey or Newman-Keuls post-*hoc* tests (*p* < 0.05). (C) The inhibitory effect of Shh antagonist cyclopamine (Cycl) is shown in Shh-LTII cells cultured with hrShh and Purm and developed with Promega as described in detail. Data are expressed as mean ± SD and shown as AU with significance value illustrated. (D) The inhibitory effect of ALS CSF shown in (B) is expressed as fold increase over baseline for each sample (AU stimulated/AU unstimulated), demonstrating a significantly increased luminescence in response to rShh and Purm treatment in presence of CSF from controls, but not ALS patients. Data analysis was performed using one way ANOVA with Tukey post-*hoc* test. Displayed *p* values indicate significant differences between groups, and N.S. indicates non-significant differences. (E) The inhibitory effect of ALS CSF observed in Shh-LTII cells (B, C) was repeated in NSC-34-Gli cells cultured in half-diluted CSF from controls and ALS patients. Data are expressed as mean ± SD and shown as AU with significance indicated on a graph. (F) Nuclear expression of Gli1, Sufu, and Gli2 proteins is shown in immunofluorescently stained NSC-34-Gli cells cultured for 72 hours in a serum-reduced medium with a half-diluted pooled CSF from Neuro and ALS groups. Data are expressed as mean ± SEM, with significance value included, and shown as a relative nuclear intensity analyzed per cell (ImageJ) over manually contoured nuclear (DAPI) area. Reduced Gli1 and Sufu nuclear translocation in ALS CSF-treated cells are shown, in contrast to elevated Gli2 nuclear expression. (G) Representative immunofluorescence images demonstrating Gli1 (green), Sufu (red), and Gli2 (red) distribution in NSC-34-Gli cells treated either with CSF from neurological controls (Neuro panel) or ALS group (ALS panel) using DAPI (blue) as a nuclear stain. Single or merged images are shown with a scale bar depicted in a bottom right image.

After determining that Shh protein levels are comparable between CSF from ALS patients and controls, we next investigated whether the biological activity of Shh signaling in ALS CSF is altered. To test this, we used an *in vitro* Shh-LTII cell assay. We established a dose-response curve using rShh (grey bar) and Purm (black bar) in culture medium (Fig 2A), and found that the lowest concentration of rShh stimulating the Shh-LTII cells above background was 0.01 μg/ml, which approximated our measured CSF levels of Shh (about 15,000 pg/ml or 0.015 μg/ml). Thus, the Shh-LTII cell assay lacks sensitivity to directly test for CSF Shh biological activity.

However, an inhibitory action of ALS CSF was readily apparent when fixed concentrations of rShh (0.3 μg/ml) were added to CSF in the assay (Fig 2B). In both normal controls and neurological controls, added rShh increased induced luminescence as expected. In contrast, there was no stimulatory effect seen with rShh added to ALS CSF (Fig 2B, grey bar). Note that the levels of RL and FFL output were comparable between the cell groups before the addition of rShh (RL output (mean ± SD): 71.69 ± 27 for controls and 53.78 ± 20.05 for ALS group, *p* = 0.4058; FFL output: 60.69 ± 14.09 for controls and 61 ± 18.29 for ALS group, *p* = 0.9649). These results suggest that the inhibitory effect observed in ALS CSF-treated cells was not due to decreased metabolic activity or survival of these cells compared to control cells.

We repeated the assay with added Purm (0.3 μM) instead of rShh, since Purm is a Shh agonist acting downstream of Shh. As with rShh, Purm stimulates light output in normal and neurological controls CSF but not with ALS CSF (Fig 2B, black bar). The addition of cyclopamine (5μM), which blocks Purm binding to Smo [51], blunts the response of Purm added to the culture medium (AU for rShh alone 2.19 ± 0.49 (mean ± SD), for rShh + Purm 3.95 ± 0.33, and for rShh + Purm + cyclopamine 2.51 ± 0.6, *p* = 0.0018 for rShh + Purm versus rShh + Purm + cyclopamine), confirming that the effect of rShh and Purm in Shh-LTII cells goes through the canonical pathway and is Smo-dependent (Fig 2C). Collectively, these data indicate an inhibitory activity of ALS CSF, but not control CSF, that interferes with Shh functional activity and downstream Gli-dependent signaling below the level of Smo. As an alternative presentation of an inhibitory effect of CSF on Shh action in Shh-LTII cells, data were quantified and expressed as fold increase over baseline (AU stimulated/AU unstimulated). Here as well, rShh and Purm stimulated light output in normal and neurological controls CSF, but not with ALS CSF (Fig 2D), illustrating compromised Shh signaling in ALS CSF samples.

The inhibitory activity of ALS CSF on Shh signaling was also confirmed in NSC-34-Gli cells. These cells represent a mouse neuroblastoma/embryonic spinal cord hybrid cell line enriched for motor neurons [48] that were subsequently transfected to express luciferase under Gli regulation as described in Materials and Methods, thus the name NSC-34-Gli (Fig 2E). In agreement with results from Shh-LTII cells, the luminescence output was also significantly reduced in ALS CSF-treated NSC-34-Gli cells, compared to light output from control cells (*p* = 0.0412) (Fig 2E) indicating that this ALS CSF-driven inhibition is not restricted to a single cell type.

Furthermore, with the goal of determining downstream events and factors associated with the ALS CSF-driven inhibitory effect, we analyzed the nuclear distribution of Gli1, Gli2, and Sufu proteins in immunofluorescently stained NSC-34-Gli cells cultured with pooled CSF from ALS and Neurological control groups. The analysis was performed by quantifying the intensity of the stain of interest in the nuclear area defined in a DAPI channel. The analysis indicated that ALS CSF-driven reduction in luminescence in NSC-34-Gli cells (Fig 2E) was associated with significantly reduced nuclear intensity of Sufu (34.13 ± 8.55, n = 520 in controls versus 28.44 ± 5.63, n = 429 in ALS group, *p* < 0.0001, t-test) (Fig 2F), suggesting its potential entrapment in the cytoplasm in complex with other factors such as Gli1, as reported before [52]. Our data showing that nuclear intensity of Gli1 was also reduced in ALS CSF-treated cells (24.33 ± 6.57, n = 520 in control cells versus 23.29 ± 5.67, n = 429 in ALS CSF-treated cells, *p* = 0.0044 t-test), but not Gli2 (12.74 ± 2.27, n = 510 in controls versus 13.56 ± 3.16, n = 415 in ALS CSF-treated group, *p* < 0.0001) would support this notion (Fig 2F). Representative images depicted in Fig 2G further support aforementioned observations. Specifically, images from ALS-CSF-treated cells (ALS panel) stained for Gli1 and Sufu show more pronounced cytoplasmic and perinuclear cuffing of Gli1 (green) and Sufu (red), resulting in orange color present in the cytoplasmic area in the merged Gli1/DAPI/Sufu channel. In contrast, little or no orange and lighter green (due to overlapping green (Gli1) and blue (DAPI) staining in Neuro-CSF-treated cells (Neuro panel) is evident, where Gli1 and Sufu were more noticeably present within the nuclear area. Staining for Gli2, however, revealed more pronounced Gli2 presence in the nuclear area in ALS panel, compared to controls. These differential patterns of Gli1, Sufu, and Gli2 distribution were unlikely due to altered overall levels of these factors, since no significant differences were found in total (cytoplasmic and nuclear) levels of expression of Gli1 (*p* = 0.3899, unpaired two-tailed t test), Gli2 (*p* = 0.1180), and Sufu (*p* = 0.058) between controls and ALS-CSF-treated cells, suggesting that these alterations in nuclear translocation likely reflect changes in functional activity of Shh pathway triggered by CSF stimulation.

### TNF-α and IL-10 are increased in ALS CSF

Oxidative damage and neuroinflammation play a significant role in neurodegeneration observed in ALS, and IFN-γ and TNF-α are prominent contributors to these processes. Levels of both of these cytokines have been reported elevated in patients with ALS [46, 53]. Based on earlier observations showing the involvement of IFN-γ in the paradoxical dysregulation of Shh-Gli1 pathway in neural stem cells in a model of autoimmune encephalomyelitis [54], and that TNF-α and IFN-γ synergistically induce inflammation, oxidative damage, and motor neuron apoptosis in rat spinal cord embryonic explants [55], we hypothesized that the inhibitory effect observed in our Shh-LTII assay could be related to elevated IFN-γ and TNF-α, and/or other cytokines present in the CSF of ALS patients. To test this, we first measured levels of 14 different biological analytes (cytokines, chemokines, growth and differentiation factors), including IFN-γ, by multiplex assay or ELISA (Table 2). As might be expected, there is considerable variability in some of these values, especially for neurological controls with a wide range of underlying pathologies. Values of two analytes were significantly increased,–both in ALS CSF: pro-inflammatory TNF-α and anti-inflammatory IL-10 (Table 2), and several others were elevated but did not reach significance. Levels of IFN-γ were highly variable in neurological controls, and in some instances much higher than in ALS patients.

**Table 2:** Levels of analytes (pg/ml) measured in the CSF of normal controls (n = 13), neurological controls (n = 12), and patients with ALS (n = 9).

Analytes	Normal controls	Neurological controls	ALS	*p* value
**GDF11**	37.05 ± 14.52	32.98 ± 17.56	30.00 ± 19.87	0.346
**GM-CSF**	0.00 ± 0.00	0.77 ± 2.65	0.10 ± 0.20	0.091
**IFN γ**	0.70 ± 0.20	35.21 ± 118.93	0.87 ± 0.31	0.115
**IL-1β**	1.30 ± 1.80	1.65 ± 2.44	0.75 ± 0.93	0.411
**IL-6**	1.23 ± 2.60	17.72 ± 37.95	0.24 ± 0.65	0.278
**IL-8**	25.71 ± 15.43	253.77 ± 658.53	20.55 ± 7.12	0.362
**IL-10**	1.09 ± 0.38	16.53 ± 50.93	1.48 ± 0.47	0.045 *
**IL-17A**	0.02 ± 0.04	0.02 ± 0.04	0.22 ± 0.38	0.074
**IP-10**	391.96 ± 288.08	4918.58 ± 11158.42	288.76 ± 71.92	0.114
**MCP-1**	886.58 ± 607.67	978.16 ± 799.52	1014.98 ± 322.62	0.570
**MIP-1β**	3.51 ± 2.64	4.99 ± 4.53	3.08 ± 1.78	0.663
**sTLR2**	17.46 ± 49.62	165.73 ± 436.61	1.82 ± 3.64	0.360
**TNF-α**	7.95 ± 5.54	9.72 ± 7.53	13.48 ± 5.56	0.033 *
**VEGF**	83.15 ± 67.87	122.76 ± 113.67	50.21 ± 67.96	0.277

Data presented as mean ± SD. Statistical analysis was performed using unpaired t-test with * *p* < 0.05 indicating significant difference in levels between normal controls and ALS groups. Non-neurological controls (Control); neurological controls (Neuro); patients with amyotrophic lateral sclerosis (ALS); cerebrospinal fluid (CSF); Growth and differentiation factor 11 (GDF11); Granulocyte-macrophage colony-stimulating factor (GM-CSF); Interferon-γ (IFN-γ); Interleukin-1β (IL-1β); Interleukin-6 (IL-6); Interleukin-8 (IL-8); Interleukin-10 (IL-10); Interleukin-17A (IL-17A); Interferon-inducible protein-10 kilo Daltons (IP-10); Monocyte chemoattractant protein 1 (MCP-1); Macrophage inflammatory protein 1β (MIP-1β); soluble toll-like receptor 2 (sTLR2); Tumor necrosis factor-α (TNF-α); Vascular endothelial growth factor (VEGF).

### The stimulatory effect on Shh-LTII assay correlates with CSF Shh in normal controls and with CSF IFN-γ and IL-17 in ALS patients

Given the considerable variability in analyte levels, we reasoned that a correlation analysis might furnish additional information. As such, we asked whether individual levels of analytes in the CSF correlated with Shh-LTII cell output for each sample (Table 3). Shh levels strongly correlated with Shh-LTII cell stimulation in normal controls as expected, but not in ALS, also as expected. However, we did not detect a significant correlation to the Shh-Gli pathway among the other analytes examined. Indeed, contrary to what we expected, in ALS CSF but not in control CSF, there were positive correlations with IFN-γ and IL-17 (*p* < 0.005); that is, higher levels of IFN-γ were associated with greater light output. These results suggest that the inhibitory activity of ALS CSF samples was overriding the stimulatory effect of Shh, IFN-γ and IL-17.

**Table 3:** Correlation (Spearman *r)* between levels of individual analytes in CSF and luminescence measured in arbitrary units in Shh-LTII cells cultured with CSF samples and rShh.

Analytes	Normal controls	Neurological controls	ALS
**GDF11**	0.203	- 0.032	0.101
**GM-CSF**	- 0.116	0.392	0.099
**IFN-γ**	0.111	0.351	0.692 *
**IL-1β**	0.156	0.108	0.358
**IL-6**	- 0.126	0.138	0.388
**IL-8**	0.102	0.406	0.500
**IL-10**	0.095	0.298	0.093
**IL-17A**	0.448	0.043	0.699 *
**IP-10**	0.132	0.112	0.450
**MCP-1**	- 0.083	- 0.448	- 0.150
**MIP-1β**	0.027	0.082	0.035
**Shh**	0.591 *	0.007	- 0.283
**sTLR2**	0.084	- 0.004	0.365
**TNF-α**	- 0.011	0.281	0.250
**VEGF**	0.173	0.135	- 0.138
**protein**	0.479	0. 161	- 0.067

Coefficient *r* is shown (Spearman’s rank correlation), with bolded text where * *p* < 0.05. Shh-LTII cells–murine fibroblasts responsive to Shh protein by activating GLI-dependent production of a light-generating enzyme; arbitrary units (AU); luminescence light unit (LLU); firefly luciferase (FFL); Renilla luciferase (RL); AU is LLU FFL/RL;; patients with ALS (ALS); cerebrospinal fluid (CSF); Growth and differentiation factor 11 (GDF11); Granulocyte-macrophage colony-stimulating factor (GM-CSF); Interferon-γ (IFN-γ); Interleukin-1β (IL-1β); Interleukin-6 (IL-6); Interleukin-8 (IL-8); Interleukin-10 (IL-10); Interleukin-17A (IL-17A); Interferon-inducible protein-10 kilo Daltons (IP-10); Monocyte chemoattractant protein 1 (MCP-1); Macrophage inflammatory protein 1β (MIP-1β); Sonic hedgehog (Shh); soluble toll-like receptor 2 (sTLR2); Tumor necrosis factor-α (TNF-α); Vascular endothelial growth factor (VEGF).

### TNF-α is a significant inhibitor of Shh biological activity *in vitro*

**Fig 3:**
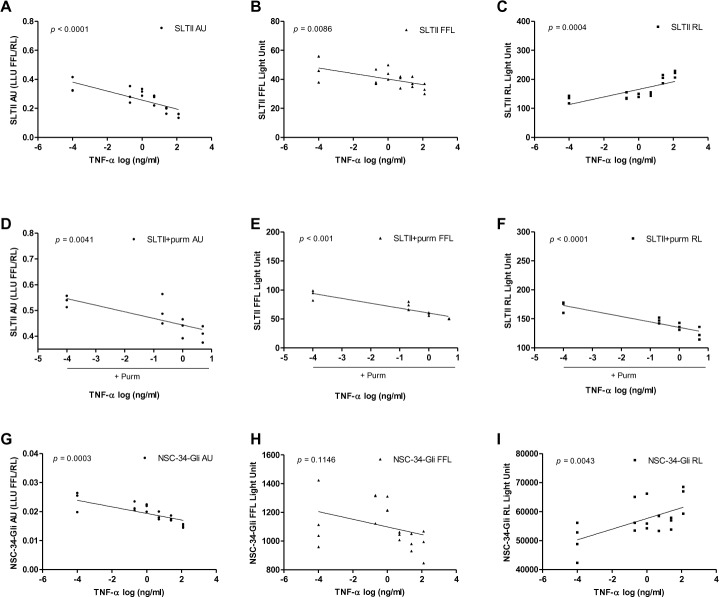
TNF-α is a significant inhibitor of Shh biological activity *in vitro*. The inhibitory effect of human recombinant TNF-α (hr TNF-α) is shown based on luminescence assay and linear regression plots are shown with corresponding *p* values. (A-C) Shh-LTII cells were cultured without or with hrTNF-α alone (0–125 ng/ml), or in the presence of Purm (0.8 μM) (D-F), and (G-I) NSC-34-Gli cells were cultured without or with hrTNF-α alone. Luminescence output was quantified 48–72 hours later with Promega. Individual data points are shown as arbitrary units (AU) (A,D,G) where FFL was normalized to RL (LLU FFL/RL), as well as raw data points for FFL(B,E,H) and RL (C,F,I).

Since levels of TNF-α were significantly elevated in ALS CSF compared to controls and since TNF-α has been implicated in the neurodegeneration, a characteristic of ALS, we sought to test whether or not TNF-α present in ALS CSF could be contributing to reduced biological activity in Shh-LTII assay. Culturing both Shh-LTII (Fig 3A–3F) and NSC-34-Gli cells (Fig 3G–3I) with human recombinant TNF-α (hrTNF-α) revealed an inhibitory effect of this cytokine on Shh signaling as the light output was significantly reduced and in a dose-dependent fashion as demonstrated in linear regression plots Fig 3A, 3D and 3G) and significant *p* values indicating a linear relationship between AU and TNF-α concentration. Presented raw data for FFL and RL indicate that TNF-α treatment preferentially inhibited FFL (Fig 3B, 3E and 3H), but not RL readings (Fig 3C, 3F and 3I), suggesting that overall inhibitory effect of TNF-α was not due to its deleterious effect on cell viability in both Shh-LTII and NSC-34-Gli cells (Fig 3A–3C and 3G–3I). Furthermore, at higher TNF-α concentration (> 25 ng/ml), RL levels were increased as depicted in Fig 3C, likely indicating a stimulatory effect of TNF-α on cellular viability and proliferation, which was expected given that both pro-and anti-apoptotic effects of TNF-α signaling have been observed as reviewed in reference [6].The TNF-α inhibitory effect remained after the addition of Shh-agonist, Purm (Fig 3D–3F). However, when TNF-α treatment was combined with purmorphamine (Fig 3D–3F), RL readings were significantly reduced with increasing TNF-α concentration. These results may suggest that when combined, TNF-α and Purm treatments could be triggering a complex cellular pro-apoptotic circuitry, perhaps as a feedback mechanisms, and thus may need to be interpreted cautiously. Our data also indicate that the lowest concentration of TNF-α inducing any meaningful decrease in light output in both cell lines is around 5–25 ng/ml, which is much higher than the concentration measured in ALS CSF (13.48 ± 5.56 pg/ml or 0.013 ng/ml), which could explain the absence of any correlation between Shh-LTII assay and TNF-α levels in CSF. Of course, tissue levels of TNF-α in the neuropil could be much higher than in the CSF.

### Shh levels and bioactivity do not correlate with disease severity or progression

Our last objective was to determine whether Shh levels or the magnitude of the Shh inhibitory activity from the ALS patients could predict ALS disease characteristics such as disease severity, duration, or progression rate. To this end, we correlated the Shh CSF levels and Shh-LTII data with ALSFRS-R score and disease duration and disease progression rate as previously defined.

Neither the CSF Shh levels nor the Shh-LTII assay data correlated with these clinical parameters (Table 4). The rate of disease progression had a strong negative correlation with ALSFRS-R score as expected (*r* = -0.797, *p* < 0.05), indicating that a lower disease progression rate is associated with higher clinical score. The GDF11 level positively correlated (*r* = 0.667, *p* < 0.05) with the ALSFRS-R, implying a higher GDF11 level predicted more favorable disease. TNF-α showed a strong inverse correlation with disease duration (*r* = -0.787, *p* < 0.05) and a positive, albeit insignificant, correlation with disease progression (*r* = 0.233, *p* > 0.05), both implying adverse disease characteristics. There was a strong negative correlation of IL-1β with disease duration (*r* = -0.698, *p* < 0.05) and a positive, but insignificant, correlation with disease progression (*r* = 0.087, *p* > 0.05), both implying adverse disease characteristics.

**Table 4:** Correlations (Spearman *r*) between levels of individual analytes in CSF of ALS group, luminescence measured in arbitrary units in Shh-LTII assay and ALSFRS-R, disease duration, and disease progression rate.

Analytes	ALSFRS-R	Disease duration	Disease progression rate
**GDF11**	0.667 *	- 0.326	- 0.354
**GM-CSF**	0.386	- 0.298	- 0.208
**IFN-γ**	0.645	- 0.503	- 0.284
**IL-1β**	0.341	- 0.698 *	0.087
**IL-6**	0.664	- 0.275	- 0.434
**IL-8**	0.321	- 0.619	0.067
**IL-10**	- 0.070	- 0.004	0.093
**IL-17A**	0.609	- 0.566	- 0.192
**IP-10**	0.139	- 0.310	0.133
**MCP-1**	- 0.468	0.259	0.233
**MIP-1β**	- 0.179	- 0.087	0.173
**Shh**	- 0.199	- 0.017	0.233
**sTLR2**	0.142	- 0.160	- 0.046
**TNF-α**	0.165	- 0.787 *	0.233
**VEGF**	0.172	0.304	- 0.385
**Disease duration**	- 0.144	NA	- 0.452
**Disease progression rate**	- **0.797** *	- 0.452	NA
**Shh-LTII assay**	0.355	- 0.628	0.083

Data analyzed with Spearman’s rank correlation and coefficient *r* is shown, with bolded text where * *p* < 0.05. Amyotrophic lateral sclerosis (ALS); revised amyotrophic lateral sclerosis functional rating scale (ALSFRS-R); cerebrospinal fluid (CSF); not applicable (NA).

## Discussion

We and others have previously demonstrated abnormalities of potential significance in the Shh signaling pathway in both *in vitro* and *in vivo* models of ALS [25, 30, 43] and in clinical samples [44]. Here, we asked whether Shh protein levels or biological activity in spinal fluid was less in ALS patients than controls, and whether these measures could be correlated with ALS disease severity, disease progression, and with other CSF analytes of biological interest in ALS.

First, CSF Shh protein levels did not differ between groups, perhaps somewhat surprisingly since Shh levels are elevated in other conditions associated with neuro-inflammation, such as multiple sclerosis and experimental allergic encephalomyelitis [54], neurodegeneration [56], and brain injury [57]. We saw no difference with neurological controls, as might perhaps be understandable, but also no difference with non-neurological controls without CNS involvement.

However, even at an early disease presentation, ALS CSF inhibits Shh signaling in both Shh-LTII and NSC-34-Gli cell-based assays. Examining Fig 2C, there is also a mild inhibition with neurological control CSF compared with non-neurological control CSF. This did not reach significance, but our numbers are small. Nonetheless, by far the most important effect is seen with ALS CSF. The level of inhibition must be below the level the Shh receptor Ptch, and below the downstream Smo, since the inhibitory effect was seen in the presence of added rShh which acts on Ptch, and with added Purm which directly activates Smo.

Shh-LTII cells are modified mouse fibroblasts and are the standard model for assessing Shh function. These cells have an internal Renilla luciferase that is constitutionally expressed under a TK promoter to control for cell viability and function, and thus our results cannot be explained on the basis of general toxicity of ALS CSF to the cells. However, our results only bear directly on ALS if Shh signaling pathways in Shh-LTII cells are similar to Shh signaling in motor neurons, although we have no reason to think otherwise. Thus, to expand and corroborate our findings from the Shh-LTII cells, we repeated main experiments using NSC-34-Gli cells, expressing a Gli- inducible firefly luciferase and a constitutively expressed Renilla luciferase. Our findings in NSC-34-Gli cells are in agreement with the Shh-LTII results and confirm an inhibitory effect of ALS CSF on the biological activity of Shh.

The mechanism of this inhibitory effect of ALS CSF on Shh biological activity is unclear. However, the reduced light output observed in NSC-34-Gli cells is associated with decreased nuclear translocation of Sufu and Gli1 and could point towards their physical interaction and entrapment in the cytoplasm, as reported before [39], which remains to be elucidated.

Oxidative damage and neuroinflammation play a significant role in neurodegeneration observed in ALS, and IFN-γ and TNF-α are prominent contributors to these processes. Elevated levels of both of these cytokines have been reported in patients with ALS [46, 53], supporting our results for TNF-α. No correlations were identified between the magnitude of the ALS CSF inhibition and biological analytes we measured in ALS CSF samples. As such, the nature of the inhibition remains speculative. Based on the involvement of IFN-γ in the paradoxical dysregulation of the Shh-Gli1 pathway in neural stem cells in a model of autoimmune encephalomyelitis [54], and the collective role of both IFN-γ and TNF-α in inducing inflammation and apoptosis in motor neurons from rat spinal cord embryonic explants, we anticipated that the implicated factors might be IFN-γ and/or TNF-α. However, in our model IFN-γ levels were not unduly increased in ALS and indeed correlated positively with the Shh-LTII assay. It is thus unlikely that the inhibition relates to IFN-γ. Moreover, despite significantly elevated TNF-α CSF levels in the ALS group, no correlation with Shh-LTII inhibition was found. Our data from direct stimulation of both Shh-LTII and NSC-34 cells with TNF-α demonstrate that TNF-α can significantly downregulate Shh signaling, an effect previously reported [58], albeit in a different cell type. However, a stimulatory effect of TNF-α on Shh signaling has also been observed [59, 60]. Altogether, these data may suggest potentially novel regulatory roles of TNF-α in Shh signaling that may warrant further investigation.

We did observe increased pro-inflammatory TNF-α and IL-17 and immune-regulatory IL-10 in ALS CSF, which is in agreement with earlier reported findings for TNF-α [61, 62], IL-10 [63], and IL-17 [46, 63–65]. We also found a negative correlation between CSF TNF-α and disease duration not previously reported, suggesting that higher levels of TNF-α were predictive of shorter disease duration and faster progression. This is not surprising since TNF-α has been shown to potentiate glutamate-mediated cytotoxicity [5, 6], a suggested mechanism of cell death in ALS. Higher CSF levels of GDF11 were associated with a better disease outcome and higher clinical score, suggesting a potentially novel role of GDF11 in ALS. Similarly to Shh, GDF11 has been shown to contribute to neuronal growth and differentiation [66–68] as well as neurogenic rejuvenation of the aging mouse brain [69].

No correlation was found between the inhibitory activity of ALS CSF in the Shh-LTII assay and measures of disease severity or progression. This may not be surprising, given the early stages of disease in the ALS patients, their clinical heterogeneity, and the scale used to estimate disease severity and progression. ALS is temporally and spatially heterogeneous, and early in the disease process there are parts of the neuraxis which are affected while most remain unaffected. Microglial cells change from a neuroprotective profile (M2) in unaffected regions to a neurotoxic (M1) profile in diseased regions, with differing patterns of cytokine production [3, 70], yet CSF derives from all regions and important correlations might be obscured. Namely, reasonably small areas of focal involvement in eloquent regions of the neuraxis, such as with bulbar involvement, might not perturb CSF cytokine measures in a major way yet might cause major functional impairment as reflected in the ALSFRS-R. Unfortunately, there are no other standardized ways of characterizing ALS extent and severity.

To conclude, this is the first study to evaluate the role of Shh levels and the biological activity in the CSF of ALS patients. There is no difference in CSF Shh levels; however, the biological activity of Shh in the ALS CSF samples is compromised and shows evidence of an inhibitor to Shh signaling that acts below the Shh receptor Ptch and below the downstream Smo, but does not correlate with any measured analyte, or with markers of disease severity. The biological significance of these observations must await further investigation.
